# Linear programming method to construct equated item sets for the implementation of periodical computer-based testing for the Korean Medical Licensing Examination

**DOI:** 10.3352/jeehp.2018.15.26

**Published:** 2018-10-18

**Authors:** Dong Gi Seo, Myeong Gi Kim, Na Hui Kim, Hye Sook Shin, Hyun Jung Kim

**Affiliations:** 1 Department of Psychology, College of Social Science, Hallym University, Chuncheon, Korea; 2 Department of Education, College of Education, Kangwon National University, Chuncheon, Korea; 3 Division of Nursing, Hallym University, Chuncheon, Korea; Hallym University, Korea

**Keywords:** Korea Medical Licensing Examination, Periodical examination, Linear programming, Predicted correct answer rate, Actual answer rate

## Abstract

**Purpose:**

This study aimed to identify the best way of developing equivalent item sets and to propose a stable and effective management plan for periodical licensing examinations.

**Methods:**

Five pre-equated item sets were developed based on the predicted correct answer rate of each item using linear programming. These pre-equated item sets were compared to the ones that were developed with a random item selection method based on the actual correct answer rate (ACAR) and difficulty from item response theory (IRT). The results with and without common items were also compared in the same way. ACAR and the IRT difficulty were used to determine whether there was a significant difference between the pre-equating conditions.

**Results:**

There was a statistically significant difference in IRT difficulty among the results from different pre-equated conditions. The predicted correct answer rate was divided using 2 or 3 difficulty categories, and the ACAR and IRT difficulty parameters of the 5 item sets were equally constructed. Comparing the item set conditions with and without common items, including common items did not make a significant contribution to the equating of the 5 item sets.

**Conclusion:**

This study suggested that the linear programming method is applicable to construct equated-item sets that reflect each content area. The suggested best method to construct equated item sets is to divide the predicted correct answer rate using 2 or 3 difficulty categories, regardless of common items. If pre-equated item sets are required to construct a test based on the actual data, several methods should be considered by simulation studies to determine which is optimal before administering a real test.

## Introduction

Until now, the Korea Medical Licensing Examination (KMLE) has been administered in the form of a paper-based test. Recently, the Korea Health Personnel Licensing Examination Institute has decided to implement a computer-based test in the near future and to employ computerized adaptive testing (CAT) in the long term. However, with computer-based exams, the number of examinees who can be tested at one time is limited. Therefore, it is preferable to introduce a periodic test in which multiple tests are administered over a certain period of time. In this context, it is necessary to construct multiple item sets and to design the tests by estimating the difficulty levels of all item sets. As a result, it is necessary to determine a statistical method to construct item sets and to equate item difficulty for effective periodical examinations.

This study aimed to identify the best method of developing an equivalent item set for the implementation of periodical computerbased testing of the KMLE. Specifically, a simulation study using linear programming (LP) was done to equate the difficulty of item sets. Furthermore, the best method to minimize the difference of the mean difficulty among 5 item sets was suggested.

## Methods

### Ethical statement

The raw data file of study was provided by the Korea Health Professional Licensing Examination Institute for research purpsoe only. There was no idenfifer of the examiness from examinee’s response file.

Therefore, informed consent and permission of institutional review board was exempted according the Korean law of “Enforcement Rule of Bioethics and Safety Act”.

### Materials and/or subjects

The average number of KMLE candidates is over 3,500 every year. Therefore, this study assumed that 5 item sets would be needed, with 1,000 students taking each set. The simulation study was conducted using 6 years of cumulative data. To validate the data, this study investigated item difficulty parameters and the ability distributions of the candidates for each year. The resulting dataset consisted of a total of 2,410 items, including 450 items from 2012, 400 items from 2013, 400 items from 2014, 400 items from 2015, 400 items from 2016, and 360 items from 2017. This study assumed that the item bank would include 2,410 items and constructed 5 item sets, each consisting of 360 items.

### Study design

The necessary constraints to construct 5 equated item sets are as follows. First, it is important to balance several content areas on the KMLE. Item sets were categorized according to the subjects of the licensing examination. The sub-factors of the KMLE are composed of 8 categories according to the subjects of the licensing examination and 18 categories according to a more specific classification. In general, if sub-factors are too specific and numerous, equating 5 item sets is inefficient and cannot be accomplished through LP because there are too many degrees of freedoms. As a result, this study sought to balance 8 sub-factors based on the subjects of the licensing examination. The DETECT value [1] was used to examine the extent of the multidimensional simple structure of the KMLE for the 6 years of cumulative data. A confirmatory DETECT analysis was conducted by using the ‘sirt’ package [2] in the R statistical software 3.4.4 (The R Foundation for Statistical Computing, Vienna, Austria) [3]. All the DETECT values were less than 0.1 (0.019 in 2012, 0.025 in 2013, 0.024 in 2014, 0.025 in 2015, 0.025 in 2016, and 0.028 in 2017). This indicates that each year of data was essentially unidimensional. The KMLE is composed of easy items, as seen by the fact that its pass rate is over 90%. For this reason, the DETECT program might provide results showing the 8-dimensional data as unidimensional. We supposed that each year of data was multidimensional based on the test specification that comprised 8 categories.

Second, the mean and standard deviation of the item difficulty statistics across the 5 item sets should be the same. Because using a constraint according to which these values had to be exactly the same would drastically reduce the amount of mathematically feasible solutions, this study implemented a constraint according to which the mean and standard deviation had to be similar across the 5 item sets. Therefore, the item difficulty statistics were divided into 2 categories and 3 categories using the predicted correct answer rate (PCAR), and the same number of items was assigned for each item difficulty category.

In this case, the item difficulty was determine using the PCAR, which was computed by the KMLE item developers when they created items and could be interpreted as a predicted value. The PCAR ranged from 0 to 100, with values interpreted as the ratio of the number of correct responses to the total number of responses. If the PCAR is large, the item is easy, and vice versa.

Previously, the PCAR was divided into 6 categories based on the subjects of the licensing examination to determine the difficulty constraint. The item parameter distribution of the PCAR is presented in Table 1.

Almost 90% of PCARs were between 60 and 90. Based on a previous investigation [4], it is meaningless to divide the PCAR into 6 categories. Two categories divided by a PCAR of 75 or 3 categories divided by PCARs of 60 and 75 would be appropriate for setting equal item difficulty constraints. As a result, this study examined the quality of equating 5 item sets using 2 or 3 divisions of item difficulty. Based on this item difficulty design, this study examined which equating conditions provided 5 equally pre-equated item sets.

Third, this study investigated whether common items could contribute to the accuracy of equating of item sets. Item sets that had 20% of the total items in common and item sets without common items were constructed and compared with each other.

This study was designed through the following procedures. First, 5 item sets were constructed by LP using 2 or 3 divisions of the PCAR, and then compared with 5-item sets constructed by random item selection. Second, 5 item sets with 20% of the items in common were compared with 5 item sets without common items.

To compare the accuracy of equating in each condition, we estimated the actual correct answer rate (ACAR) and the difficulty parameter of item response theory (IRT). The Rasch model was used to estimate the IRT difficulty parameters in this study [5]. In the Rasch model, we used the marginal maximum likelihood method for item parameter estimation and the expected a posterior for ability parameter estimation. IRT difficulty parameters were estimated on the assumption that the candidates’ abilities were the same every year. Therefore, the ACAR and IRT difficulty parameters were used to evaluate the equating accuracy.

### Technical information

To equate the difficulty of the item sets, this study conducted a simulation study using LP, as suggested by van der Linden [6]. Each item set was composed of 360 items from the item bank. The item bank consisted of 8 sub-factors to consider the content balancing issue [7]. As shown in Table 1, the sub-factors of the item bank had 317, 316, 317, 182, 958, 152, 120, and 48 items, respectively, and each sub-factor was demonstrated to be a unidimensional trait [8]. The item sets also were composed of conditions with 20% common items or without common items. The constraints can be summarized as follows: (1) generate 5 item sets; (2) the number of items in each item set is 360; (3) eight sub-factors have 45, 45, 45, 25, 154, 20, 20, and 6 items, respectively; (4) and no common items or 20% common items.

To construct an optimal test using LP, the above constraints must be transformed into decision variables and then converted to a mathematical optimization problem. Decision variables can be defined as variables that make the best decision in the optimization problem. The solution of the problem is to find a set of values such that an objective function is optimal and all constraints are satisfied [4].

The constraints of this study can be solved by selecting the following variables. i= 1,…,360 is the number of items in each item set. It is assumed that the sub-factor A1 is composed of i= 1,…,45, A2 is i= 46,…,90, A3 is i= 91,…,135, A4 is i= 136,…,160, A5 is i= 161, …,314, A6 is i= 315,…,334, A7 is i= 335,…,354, and A8 is i= 355, …,360.

The decision variable of this study was determined by a binary response for each item. If the item i is selected, *x_i_*= 1, and if the item i is not selected, *x_i_*= 0. The sum of the number of items for each item set is expressed as follows $\sum_{i=1}^{360}\chi_{i}$.

In order to equate the item sets, the average PCAR was used in this study. *P_i_* indicates the PCAR of item i. If *x_i_*= 1 is the selected item, $\sum_{i=1}^{360}P\left(\chi_{i}\right)$ is the sum of the PCARs. If $\sum_{i=1}^{360}P\left(\chi_{i}\right)$ is divided by 360, it becomes the average PCAR. This study was designed to control the average PCARs of each item set as closely as possible. The difference in average PCARs should be smaller than τ= 0.05. The LP that obtains the optimal test from these constraints is summarized as follows.

(1) The number of item sets is k = 5.

(2) For all k, the average PCARs expressed by $\frac{\sum_{i=1}^{360}P\left(\chi_{i}\right)}{360}$ are similar.

(3) The number of items for the 8 sub-factors in each item set is as follows.

$$\sum_{i=1}^{317}\chi_{i}=45\;,\;\sum_{i=318}^{633}\chi_{i}=45\;,\;\sum_{i=634}^{950}\chi_{i}=45\;,\;\sum_{i=951}^{1132}\chi_{i}=25\;,\;\;\sum_{i=1133}^{2090}\chi_{i}=154\;,\;\sum_{i=2091}^{2242}\chi_{i}=20\;,\;\sum_{i=2243}^{2362}\chi_{i}=20\;,\;\sum_{i=2363}^{2410}\chi_{i}=6\;$$

(4) The no-common-items constraint is defined as $\sum_{k=1}^{5}\chi <1,\;for\;all\;i.$

(5) The common-items constraint is defined as $\sum_{k=1}^{5}\chi \leq n_{0}^{max},\;for\;all\;i\;\left(n_{0}^{max}>1\right)$

The objective function of (2) is to create 5 item sets that are equated. The constraint in (2) mandates that the 5 item sets have a difference of the average PCAR of 0.05 or less. The constraint in (3) determines the number of items for the 8 sub-factors in each item set. The constraint in (4) expresses the absence of common items, while (5) formalizes the presence of common items among the 5 item sets.

The relationship between all constraints was linear. Therefore, the design for this study is equivalent to an LP for 0–1. The solution of an LP is to have 5 item sets that equate to a considerable degree. The solution is determined by a 0–1 value for which the objective function is minimal, and all constraints are met under the appropriate conditions. To summarize, this study was designed to construct 5 item sets that were equated from the item bank.

### Statistical analysis

The various conditions were compared in terms of PCAR, ACAR, and IRT difficulty parameters. The random equating method using PCARs was compared with the pre-equating method using LP with 2 or 3 PCAR categories. In addition, the equating condition with common items was compared to the condition without common items. Finally, analysis of variance (ANOVA) was implemented for the dependent variables of ACAR and the IRT difficulty to determine whether there was a significant difference among the different equating conditions. This study used R statistical software (The R Foundation for Statistical Computing) for all statistical analyses [3]. The R code for constructing item sets using LP is presented in Appendix 1.

## Results

The results of the pre-equating of 5 item sets are presented in Table 2. In Table 2, the degree of equivalence of 5 item sets is compared using the LP method. The dependent variables are the average of ACAR, PCAR, and IRT difficulty. To statistically confirm the preequating of the 5 item sets under each condition, ANOVA was performed, and the results are presented in Table 3.

There was a statistically significant difference in IRT difficulty when 5 item sets were composed randomly without common items (F(4,179) = 4.075, P= 0.003, η^2^= 0.010). This means that the 5 item sets were not equated. There was no statistically significant difference between ACAR and IRT difficulty when PCAR was divided into 2 or 3 categories, regardless of the presence of common items. In other words, when the PCAR was divided into 2 or 3 categories, it was verified that the mean ACAR and IRT difficulty among the 5 item sets were similar to each other.

In the comparison of the common-item and no-common-item conditions, the majority of P-values in the evaluation criteria were above the 0.05 significance level. This means that there were no significant differences among the item sets.

## Discussion

Using real data, this study proposed the use of LP to construct 5 item sets that reflected the characteristics of each content area. It can be seen that the use of common items did not significantly contribute to the equating of the 5 item sets (Tables 2, 3). It is recommended that common items should not be used to equate 5 item sets because of item exposure.

LP performed well for constructing 5 pre-equated item sets compared to the random and subject-based methods (Tables 2, 3). It was demonstrated that the LP method with real data was applicable to construct pre-equated item sets for the KMLE.

Based on this real data simulation study, several suggestions for constructing the equated 5 item sets can be made, as follows.

First, this study proposed equating 5 item sets using LP. However, an item bank must be quite large to construct a test with LP. An item bank is required to contain at least 10 to 30 times the number of items in the item sets. Even though the KMLE contains items accounting for 18 times the length of the item set, there is some pressure to produce the necessary items every year because the items of the KMLE have been released every year. To decrease this pressure, it has been suggested to reduce the number of items in each item set or to use CAT, which will be an effective alternative for future licensing examinations. CAT is advantageous because it provides only the appropriate items for each individual and estimates each candidate’s ability based on adaptive items [9]. Therefore, it is possible to reduce the burden of developing many items every year.

Second, this study showed that dividing PCAR into 2 or 3 categories is enough to equate 5 item sets. If the item bank is large enough to be divided into numerous sections according to PCAR, the item sets constructed by LP are almost perfectly equated.

Third, the results using PCAR and ACAR criteria in empirical data conducted from 2012 to 2017 showed a small difference (for 2012 to 2017, the average ACAR was 74.76 and the average PCAR was 76.89) in the KMLE (Table 2). However, if the difference is large, an alternative method should be considered to construct the parallel test. For example, it is possible to develop items with similar content for sub-factors or to construct item sets using sequential sampling. In order to reduce the difference between the PCAR and ACAR, the accuracy of the PCAR can be improved by continuously obtaining feedback on the actual difficulty after the test is taken every year.

Fourth, if pre-equating is not possible, post-equating can be considered for the real test. Post-equating means linking the item parameters of the unique items of item set A to item set B using a regression of common items [10]. Recent studies have shown that post-equating can be conducted using a concurrent calibration method [11,12]. The concurrent calibration based on IRT reduces the risk of item exposure because no common item is required. However, if item sets are equated on periodical examinations, post-equating using common items will be accurate and stable for the real test.

Fifth, when constructing an item set, the ratio of the number of items of each sub-factor must be considered through a job analysis. If sub-factors are too specific and numerous, constructing equated item sets using LP will be inefficient and will not converge because too many degrees of freedom are present. Therefore, this study proposes a job analysis to accurately estimate the appropriate number of sub-factors and to decide the composition of details.

In this study, a simulation study was conducted once under each condition. This is a limitation of this study, because it does not guarantee replication with the PCAR in actual use. In the item bank with 2,410 items, LP was used to construct 5 item sets with 360 items. For this reason, the average of the item parameters remained almost invariable even after several repetitions. In future studies, the results of this study should be confirmed by determining whether the results can be replicated using LP with a large item bank with varied conditions.

In addition, this study did not consider the problem of item difficulty parameter drift from item exposure, even if 5 item sets are constituted by the LP method. Therefore, a strategy to control item exposure is to delete items or modify parameters by analyzing item parameter drift. If item sets are developed every year for the item bank, an item monitoring system should be established to control item parameter drift from item exposure [13]. If difficulty parameter drift is detected, a small difficulty drift can be adjusted by calculating the average of 2 difficulty parameters, and if the difficulty parameter drift is large, it is desirable to eliminate items.

In conclusion, the LP method is applicable to construct equated item sets that reflect each content area. The best method to construct equated item sets suggested is to divide the PCAR into 2 or 3 difficulty categories regardless of the presence of common items. If preequated item sets are necessary to construct a test based on the actual data, several methods should be considered by simulation studies to determine which is optimal before administering a real test. For example, some potentially appropriate methods include sequential sampling of the test, concurrent calibration equating using IRT, and CAT. However, it is difficult to resolve the problem of item difficulty parameter drift from item exposure, even when using LP or other statistical methods. Therefore, if item exposure causes the item parameter drift issue, it is recommended to delete problematic items or to modify the corresponding parameters in a real examination situation.

## Figures and Tables

**Table 1.** Item distribution divided into 6 PCAR sections based on 8 subject areas

Subject area	PCAR ≤ 30	30 < PCAR ≤ 45	45 < PCAR ≤ 60	60 < PCAR ≤ 75	75 < PCAR ≤ 90	90 < PCAR ≤ 100	Total
A1	0	4	48	112	143	10	317
A2	0	0	3	68	241	4	316
A3	0	1	28	140	147	1	317
A4	0	0	14	56	110	2	182
A5	1	1	95	404	444	13	958
A6	0	0	17	47	88	0	152
A7	0	1	19	29	57	14	120
A8	0	0	3	17	28	0	48
Total	1	7	227	873	1,258	44	2,410

PCAR, predicted correct answer rate.

**Table 2.** Comparison of ACAR, IRT difficulty, and PCAR among the 5 item sets assembled by linear programming

Evaluation criteria	PCAR section	No common items					20% Common items				
		Set 1	Set 2	Set 3	Set 4	Set 5	Set 1	Set 2	Set 3	Set 4	Set 5
ACAR	Random	74.75	74.99	75.23	76.42	76.99	74.12	76.36	76.41	77.12	76.99
	2	73.65	76.12	74.20	73.93	76.31	72.79	74.72	76.13	76.20	76.05
	3	77.38	75.42	77.02	75.63	75.53	74.65	77.04	77.44	77.05	76.88
IRT difficulty	Random	–1.40	–1.63	–1.67	–1.84	–1.79	–1.62	–1.84	–1.84	–1.80	–1.80
	2	–1.54	–1.56	–1.56	–1.60	–1.72	–1.49	–1.68	–1.79	–1.75	–1.75
	3	–1.81	–1.65	–1.71	–1.68	–1.61	–1.69	–1.82	–1.85	–1.84	–1.76
PCAR	Random	76.44	77.58	78.02	77.07	78.25	77.78	77.04	77.06	78.20	78.25
	2	76.56	76.08	76.90	76.58	76.71	76.19	76.26	76.68	76.91	76.94
	3	78.33	78.13	78.14	78.22	78.27	78.02	78.34	78.45	78.26	77.98

ACAR, actual correct answer rate; IRT, item response theory; PCAR, predicted correct answer rate.

**Table 3.** Analysis of variance results of ACAR, IRT difficulty, and estimated PCAR for the 5 item sets

Evaluation criteria	PCAR section	Group	No common item					20% Ccommon items				
			SS	df	F	η^2^	P-value	SS	df	F	η^2^	P-value
ACAR	Random	B^a)^	1,377	4	0.721	0.002	0.578	2,117	4	1.115	0.002	0.348
		W^b)^	857,108	1,795				852,224	1,795			
	2	B	2,318	4	1.172	0.001	0.321	3,095	4	1.574	0.003	0.179
		W	888,050	1,795				882,320	1,795			
	3	B	1,248	4	0.644	0.003	0.631	1,787	4	0.98	0.005	0.417
		W	869,796	1,795				818,104	1,795			
IRT difficulty	Random	B	43	4	4.075	0.010	0.003^**^	12	4	1.173	0.003	0.321
		W	4,713	1,795				4,698	1,795			
	2	B	8	4	0.723	0.001	0.576	20	4	1.926	0.004	0.104
		W	4,871	1,795				4,767	1,795			
	3	B	8	4	0.763	0.001	0.549	6	4	0.6	0.001	0.662
		W	4,827	1,795				4,593	1,795			
PCAR	Random	B	784	4	2.162	0.005	0.049^**^	507	4	1.423	0.003	0.224
		W	162,682	1,795				159,893	1,795			
	2	B	132	4	0.352	0.001	0.843	181	4	0.51	0.001	0.728
		W	168,373	1,795				159,302	1,795			
	3	B	10	4	0.027	0.001	0.999	59	4	0.165	0.001	0.956
		W	163,372	1,795				160,652	1,795			

ACAR, actual correct answer rate; IRT, item response theory; PCAR, predicted correct answer rate; SS, sum of squares; df, degrees of freedom.

a) Between groups.

b) Within groups.

** P<0.05.

## Supplementary materials

Supplement 1. Audio recording of the abstract.
