# Supplementary material for: Linear programming method to construct equated item sets for the implementation of periodical computer-based testing for the Korean Medical Licensing Examination
Source: J Educ Eval Health Prof. 2018 Oct 18;15:26. doi: 10.3352/jeehp.2018.15.26 (PMC6318553; doi:10.3352/jeehp.2018.15.26)
Supplement: Supplementary file 2 [file jeehp-15-26-app1.pdf]

Appendix 1. R code for constructing item sets using linear programming

```
#################################################################
##
require(ltm)
require(irtoys)
require(catR)
require(mirt)
require(stringr)
require(irtoys)
library(lpSolveAPI)

setwd("H:\\Item_Set_Equating_2018\\data_2015_2017")
responses <- read.table("data2017.txt", header=F)
items <- read.table("itemtotal_2012_2017.csv", header=T, sep=",")
# Change the table into a numeric mode
res <- as.matrix(responses[,-1])
head(res)

#################### difficulty equating ############################
attach(items)
items$level[items$ratio<=75]<-1
items$level[items$ratio>75]<-2
#################################################################
##
##          Linear          Programing          &          5          forms
#################################################################
##
```

```
ID<-items$ID
B=items$ratio
Content=items$content
level=items$level
I=nrow(items)
F=5 ##5 forms
N=360
Vc=list()
for(k in 1:8){
Vc[[k]] = c(1:I)[Content==k]}
######################## difficulty equating
##############################
DIF=list()
for(k in 1:2){
DIF[[k]]=c(1:I)[level==k]
M=I*F+1
#Create the Model: model object with 0 constraints and 331 decision variables
lprec=make.lp(0,M)
# Set control parameters: minimization problem;
#absolute MIP gap is set to 0.1; relative MIP gap is set to 0.05
lp.control(lprec,sense="min",epsint=0.1,mip.gap=c(0.1,0.05));
## Constraints (8) and (9)
set.type(lprec,columns=c(1:(5*I)),type="binary") ## 5 forms
set.type(lprec,columns=M,type="real")
set.bounds(lprec,lower=rep(0,M),upper=rep(1,M))
#Constraint (5) No Item overlap between two forms
for(k in 1:I){
```

```
add.constraint(lprec,rep(1,5),"<=",1,indices=c(k,I+k,2*I+k,3*I+k,4*I+k)) } ## 5 forms
#Constraint (5) 30 Item overlap between two forms
#for(k in 1:I){
#add.constraint(lprec,c(1,1,1,1,1),"<=",2,indices=c(k,I+k,2*I+k,3*I+k,4*I+k))
#}
#Constraint(6)
Nc=c(45,45,45,25,154,20,20,6)
#Nc=c(4,4,4,2,10,2,2,2)

#Form 1
for (k in 1:8){
add.constraint(lprec,rep(1,length(Vc[[k]])),">=",Nc[k],indices=Vc[[k]])
}
#Form 2
for(k in 1:8){
add.constraint(lprec,rep(1,length(Vc[[k]])),">=",Nc[k],indices=I+Vc[[k]])
}
#Form 3
for (k in 1:8){
add.constraint(lprec,rep(1,length(Vc[[k]])),">=",Nc[k],indices=2*I+Vc[[k]])
}
#Form 4
for(k in 1:8){
add.constraint(lprec,rep(1,length(Vc[[k]])),">=",Nc[k],indices=3*I+Vc[[k]])
}
#Form 5
for (k in 1:8){
```

```
add.constraint(lprec,rep(1,length(Vc[[k]])),">=",Nc[k],indices=4*I+Vc[[k]])
# Constraint(7)
add.constraint(lprec,rep(1,I),"=",N,indices=1:I)
add.constraint(lprec,rep(1,I),"=",N,indices=(I+1):(2*I))
add.constraint(lprec,rep(1,I),"=",N,indices=(2*I+1):(3*I))
add.constraint(lprec,rep(1,I),"=",N,indices=(3*I+1):(4*I))
add.constraint(lprec,rep(1,I),"=",N,indices=(4*I+1):(5*I))
#Constraint(3) and (4)
Nd=c(180,180)
# form 1
for(k in 1:2){
add.constraint(lprec,rep(1,length(DIF[[k]])),">=",Nd[k],indices=DIF[[k]])
}
# form 2
for(k in 1:2){
add.constraint(lprec,rep(1,length(DIF[[k]])),">=",Nd[k],indices=I+DIF[[k]])
}
# form3
for(k in 1:2){
add.constraint(lprec,rep(1,length(DIF[[k]])),">=",Nd[k],indices=2*I+DIF[[k]])
}
#form4
for(k in 1:2){
add.constraint(lprec,rep(1,length(DIF[[k]])),">=",Nd[k],indices=3*I+DIF[[k]])
}
#form 5
for(k in 1:2){
```

```
add.constraint(lprec,rep(1,length(DIF[[k]])),">=",Nd[k],indices=4*I+DIF[[k]])

}

#Objective function

set.objfn(lprec,1,indices=M)

#Solve the model

res_flag=solve(lprec)

res_flag

x_opt=get.variables(lprec)

######################## construction 5 item sets ########################

f1<-x_opt[1: 2410]

f2<-x_opt[2411:4820]

f3<-x_opt[4821:7230]

f4<-x_opt[7231:9640]

f5<-x_opt[9641:12050]

form1<-cbind(items,f1)

form2<-cbind(items,f2)

form3<-cbind(items,f3)

form4<-cbind(items,f4)

form5<-cbind(items,f5)


form1_data<-form1[which(form1$f1==1),]

form2_data<-form2[which(form2$f2==1),]

form3_data<-form3[which(form3$f3==1),]

form4_data<-form4[which(form4$f4==1),]

form5_data<-form5[which(form5$f5==1),]


rbind(apply(form1_data,2,mean),
```

```
apply(form2_data,2,mean),
apply(form3_data,2,mean),
apply(form4_data,2,mean),
apply(form5_data,2,mean))

rbind(apply(form1_data,2,length),
apply(form2_data,2,length),
apply(form3_data,2,length),
apply(form4_data,2,length),
apply(form5_data,2,length))

table(form1_data$content)
table(form2_data$content)
table(form3_data$content)
table(form4_data$content)
table(form5_data$content)
table(table(c(form1_data$id,form2_data$id,form3_data$id,form4_data$id,form5_data$id)) )
################################# END ######################
```
